# Association of genetic polymorphisms in *IL-1R1* and *IL-1R2* genes with IgA nephropathy in the Han Chinese population

**DOI:** 10.18632/oncotarget.16929

**Published:** 2017-04-07

**Authors:** Maowei Xie, Daofa Zhang, Yin Zhang, Xiaohong Yang, Yan Su, Yanni Wang, Haiyang Huang, Hui Han, Wenning Li, Keying Fu, Huiluan Su, Wentan Xu, Jiali Wei

**Affiliations:** ^1^ Department of Nephrology, Hainan General Hospital, Haikou Hainan 570311, China; ^2^ Central Laboratory, Hainan General Hospital, Haikou Hainan 570311, China

**Keywords:** IgA nephropathy, *IL-1R1*, *IL-1R2*, genetics polymorphism, Chinese Han population

## Abstract

**Aim:**

IgA nephropathy (IgAN) is the major cause of end-stage renal disease(ESRD) in Asia and its pathogenesis is influenced by both genetic and environmental factors. Single nucleotide polymorphisms (SNPs) in *IL1R1* and *IL-1R2* may be associated with susceptibility to IgAN. In this study, we study the association between genetic variants of *IL-1R1* and *IL-1R2* and IgA nephropathy risk in the Chinese Han population.

**Result:**

In the allelic model analysis, the rs10490571 and rs3917225 were associated with a 1.40-fold, and 1.31-fold increased risk of IgA nephropathy, respectively. In the genetic model analysis, the rs10490571 in *IL1R1* was associated with a 1.46-fold increased risk of IgAN in the dominant model and 1.36-fold increased risk in the Log-additive model, respectively. However, the rs3218977 in *IL1R2* was associated with a 0.71-fold decrease risk of IgAN in the dominant model and a 0.71–fold decrease risk in the over-dominant model, respectively. We found four SNPs (rs11674595, rs4851521, rs719250, and rs3218896) constructed four haplotypes in the IL1R2 gene and none of the haplotype was significantly associated with risk of IgAN.

**Materials and Methods:**

A case-control study was conducted including 426 nephropathy patients and 463 healthy controls. Chi-squared tests and genetic model were used to evaluate associations.

**>Conclusions:**

These findings suggested that *IL-1R1* and *IL-1R2* polymorphisms may contribute to the development of IgAN.

## INTRODUCTION

IgA nephropathy (IgAN), the most common primary glomerulonephritis in the world, is defined by the predominant IgAN deposition in the glomerular mesangium [1]. The disease mainly in the upper respiratory tract infection and the course of disease may be more severe in individuals of Asian ancestry [2]. the patients with IgAN, the mesangium IgA deposits are just because of the IgA1 subclass and often show with abnormal glycosylation [3, 4], which often accompany with an upper respiratory tract infection, and about 40% of these patients will develop into end-stage renal disease within the next 20 years [5]. Great efforts have been applied to the diagnosis, monitoring and treatment of the disease. In recent years, transformative and intersecting advances have witnessed in our understanding of the biology, aetiology, and pathology of IgAN. However, the specific pathogenesis of IgAN has not yet fully state, but there is evidence that the risk factors are associated with environmental and genetic play vital roles in the etiology of IgAN [6]. Several evidence support the genetic risk factors, containing differences in ethnic, distributions in region, and individual variation in the course and prognosis of disease [7, 8].

interleukin-1 (IL-1) is a multifunctional proinflammatory cytokine that can be produced by many cell types, including monocytes, activated macrophages, and endothelial cells [9], it plays a key role in autoimmune and inflammatory diseases by activating the expression of genes associated with the innate and adaptive immune response [10]. IL-1 influences both coagulation and inflammation, so some studies reported that IL-1 SNPs associate with many immune disease. For example, previous study showed that the haplotype of *IL1B*, *IL1RN*, *IL1R1*, and *IL1R2* increased the risk of venous thrombosis [11], and *IL1/IL1Ra*, *CTLA-4* and *Apo1/Fas* genes polymorphisms associate with IgA nephropathy [12]. Interleukin 1 receptor, type 1 (*IL-1R1*) and Interleukin 1 receptor, type 2 (*IL-1R2*)are cytokine receptor that belongs to the interleukin 1 receptor family, which is an important mediator involved in many cytokine induced immune and inflammatory responses [13]. Study shows that *IL1R1* and *IL1R2* gene regulate the cell metabolism and the response of immune inflammatory induce by many cytokines [14, 15]. Morever, epidemiological studies of genes affecting IgAN have been manifested that IgAN are impacted by hereditary factors.

To validate the associations between IgAN and common susceptibility loci identified in previous genome-wide association studies (GWAS) [16, 17], we conducted a comprehensive association analysis between IgAN and 11 susceptible SNPs in the *IL1R1* and *IL1R2* gene, to further clarify their potential roles in disease and reveals the association between common SNPs and IgAN risk in the Chinese Han population. The study is to evaluate a positive finding from a previous study, to provide credibility that the initial finding is valid.

## RESULTS

### Characteristics of the participants

This study involved 889 subjects, including 426 patients (278 males and 148 females; age at diagnosis: 33 ± 12.1 years) and 463 healthy controls (265 males and 198 females; age: 50 ± 11.8 years). There were statistical differences in age and sex distribution between the case and control groups (Table 1).

**Table 1:** General characteristics the of this study population

variable	cases (*n* = 426)	%	controls (*n* = 463)	%	*P* value
sex					< 0.05
Male	278	31.3%	265	29.8%	
Female	148	16.6%	198	22.3%	
Age, yr (mean ± SD)	33.2 ± 12.1		50.6 ± 11.8		< 0.001

### The associations between IL1R1 and IL1R1 SNPs and IgAN

11 tSNPs within the *IL1R1* and *IL1R2* locus were genotyped in IgAN patients and healthy controls (Table 2). Two SNPs (rs12712127 and rs3917318) were excluded for significant deviation from Hardy-Weinberg equilibrium (*p* < 0.05), A χ2 analysis revealed that rs10490571 and rs3917225 in *IL1R1* were significantly associated with increasing IgAN risk (rs10490571, OR = 1.40, 95% CI = 1.102–1.779, *p* = 0.005; rs3917225, OR = 1.31, 95% CI = 1.080–1.592, *p* = 0.006), respectively.

**Table 2:** Allele frequencies in cases and controls and odds ratio estimates for IgAN risk

SNP	Position	Gene(s)	Locus	Alleles (A/B)	MAF		HWE *P* Value	OR (95% CI)	*P* value
					case	control			
rs11674595	102610992	IL1R2	2q11.2	C/T	0.204	0.211	0.78	0.95 (0.76–1.20)	0.685
rs4851527	102622376	IL1R2	2q11.2	A/G	0.312	0.288	0.142	1.12 (0.91–1.37)	0.284
rs719250	102623718	IL1R2	2q11.2	T/C	0.314	0.313	0.746	1.01 (0.82–1.23)	0.954
rs3218896	102631652	IL1R2	2q11.2	C/T	0.157	0.157	0.596	1.00 (0.78–1.30)	0.978
rs3218977	102641201	IL1R2	2q11.2	G/A	0.216	0.252	0.175	0.82 (0.66–1.02)	0.077
rs2072472	102643019	IL1R2	2q11.2	G/A	0.198	0.206	0.672	0.95 (0.75–1.20)	0.679
rs10490571	102717337	IL1R1	2q12.1	T/C	0.217	0.165	0.867	1.40 (1.10–1.78)	0.006*
rs12712127	102726661	IL1R1	2q12.1	G/A	0.262	0.21	**0.000^a^**	1.34 (1.07–1.67)	0.01
rs956730	102758116	IL1R1	2q12.1	A/G	0.249	0.261	0.147	0.94 (0.76–1.17)	0.583
rs3917225	102769302	IL1R1	2q12.1	T/C	0.405	0.342	0.409	1.31 (1.08–1.59)	0.006*
rs3917318	102792760	IL1R1	2q12.1	G/A	0.435	0.485	**0.012^a^**	0.82 (0.68–0.99)	0.037

HWE: Hardy-Weinberg equilibrium; SNP: Single-nucleotide polymorphism; OR odds ratio; 95% CI: 95% confidence interval.

^a^Site with HWE *P* ≤ 0.05 excluded.

*p ≤ 0.05 indicates statistical significant.

### Associations between genotype frequencies and IgAN risk

As shown in Table 3, our analyses showed that the genotype “C/A-C/C” of rs10490571 in the *IL1R1* gene was associated with an increased risk of IgAN in the dominant model (adjusted OR =1.46, 95% CI, 1.03–2.07, *P* = 0.035) and log-additive model (adjusted OR=1.36,95% CI,1.01–1.83, *P* = 0.04), respectively. The genotype “G/A” of rs4851527 in the *IL1R2* gene was significantly associated with an increased risk of IgAN, based on the results from the over-dominant model (adjusted OR = 1.43; 95% CI = 1.02–2.00, *P* = 0.038). In contrast, We found that the rs3218977 in the *IL1R2* gene was significantly associated with a decreased risk of IgAN under the dominant model(adjusted OR = 0.71; 95% CI = 0.51–0.99, *P* = 0.044 for the “G/A-G/G” genotype) and over-dominant model (adjusted OR = 0.71; 95% CI = 0.50–1.00, *P* = 0.048 for the “G/A” genotype), respectively.

**Table 3:** Relationships between *IL1R1* and *IL1R2* polymorphism and IgA nephropathy risk

Gene	SNP	Model	Genotype	control	case	Before adjusted		After adjusted		AIC	BIC
						OR (95% CI)	*P*^a^	OR (95% CI)	*P*^b^		
IL1R1	rs10490571	Codominant	C/C	323 (69.8%)	258 (61.9%)	1	0.022*	1	0.11	867.4	891.3
			T/C	127 (27.4%)	137 (32.9%)	1.35 (1.01−1.81)		1.44 (1.00–2.08)			
			T/T	13 (2.8%)	22 (5.3%)	2.12 (1.05−4.29)		1.57 (0.68–3.64)			
		Dominant	C/C	323 (69.8%)	258 (61.9%)	1	0.014*	1	0.035*	865.4	884.5
			T/C-T/T	140 (30.2%)	159 (38.1%)	1.42 (1.07–1.88)		1.46 (1.03–2.07)			
		Recessive	C/C-T/C	450 (97.2%)	395 (94.7%)	1	0.061	1	0.42	869.2	888.3
			T/T	13 (2.8%)	22 (5.3%)	1.93 (0.96–3.88)		1.40 (0.61–3.23)			
		Overdominant	C/C-T/T	336 (72.6%)	280 (67.2%)	1	0.08	1	0.067	866.5	885.6
			T/C	127 (27.4%)	137 (32.9%)	1.29 (0.97–1.73)		1.40 (0.98–2.02)			
		Log-additive	---	---	---	1.39 (1.10–1.76)	0.0062*	1.36 (1.01–1.83)	0.04*	865.6	884.8
	rs3917225	Codominant	A/A	204 (44.2%)	157 (37.6%)	1	0.022*	1	0.33	868	891.9
			G/A	200 (43.3%)	182 (43.6%)	1.18 (0.89–1.58)		1.19 (0.83–1.70)			
			G/G	58 (12.6%)	78 (18.7%)	1.75 (1.17–2.60)		1.43 (0.88–2.34)			
		Dominant	A/A	204 (44.2%)	157 (37.6%)	1	0.05	1	0.2	866.5	885.7
			G/A-G/G	258 (55.8%)	260 (62.4%)	1.31 (1.00–1.72)		1.25 (0.89–1.75)			
		Recessive	A/A-G/A	404 (87.5%)	339 (81.3%)	1	0.012*	1	0.24	866.8	886
			G/G	58 (12.6%)	78 (18.7%)	1.60 (1.11–2.32)		1.31 (0.83–2.06)			
		Overdominant	A/A-G/G	262 (56.7%)	235 (56.4%)	1	0.92	1	0.68	868	887.1
			G/A	200 (43.3%)	182 (43.6%)	1.01 (0.78–1.33)		1.07 (0.77–1.50)			
		Log-additive	---	---	---	1.29 (1.07–1.56)	0.008*	1.19 (0.95–1.51)	0.14	866	885.1
IL1R2	rs4851527	Codominant	G/G	241 (52%)	195 (46.8%)	1	0.2	1	0.12	867.5	891.4
			G/A	177(38.2%)	184 (44.1%)	1.28 (0.97–1.70)		1.43 (1.01–2.03)			
			A/A	45 (9.7%)	38 (9.1%)	1.04 (0.65–1.67)		0.99 (0.55–1.79)			
		Dominant	G/G	241 (52%)	195 (46.8%)	1	0.12	1	0.088	866.9	886.1
			G/A-A/A	222 (48%)	222 (53.2%)	1.24 (0.95–1.61)		1.33 (0.96–1.86)			
		Recessive	G/G-G/A	418(90.3%)	379 (90.9%)	1	0.76	1	0.56	869.5	888.6
			A/A	45 (9.7%)	38 (9.1%)	0.93 (0.59–1.47)		0.85 (0.48–1.49)			
		Overdominant	G/G-A/A	286(61.8%)	233 (55.9%)	1	0.076	1	0.038*	865.5	884.7
			G/A	177(38.2%)	184 (44.1%)	1.28 (0.97–1.67)		1.43 (1.02–2.00)			
		Log-additive	---	---	---	1.12 (0.91–1.37)	0.29	1.14 (0.89–1.47)	0.29	868.7	887.9
	rs3218977	Codominant	A/A	264(57.1%)	263 (63.2%)	1	0.18	1	0.12	867	890.9
			G/A	163(35.3%)	126 (30.3%)	0.78 (0.58–1.04)		0.69 (0.48–0.98)			
			G/G	35(7.6%)	27 (6.5%)	0.77 (0.46–1.32)		0.81 (0.41–1.60)			
		Dominant	A/A	264(57.1%)	263 (63.2%)	1	0.066	1	0.044*	865.2	884.3
			G/A-G/G	198(42.9%)	153 (36.8%)	0.78 (0.59–1.02)		0.71 (0.51–0.99)			
		Recessive	A/A-G/A	427(92.4%)	389 (93.5%)	1	0.53	1	0.84	869.2	888.3
			G/G	35 (7.6%)	27 (6.5%)	0.85 (0.50–1.43)		0.93 (0.48–1.81)			
		Overdominant	A/A-G/G	299(64.7%)	290 (69.7%)	1	0.12	1	0.048*	865.4	884.5
			G/A	163(35.3%)	126 (30.3%)	0.80 (0.60–1.06)		0.71 (0.50–1.00)			
		Log-additive	---	---	---	0.83 (0.67–1.03)	0.089	0.79 (0.61–1.04)	0.092	866.4	885.5

SNP: Single nucleotide polymorphism; OR: odds ratio; 95% CI: 95% confidence interval.

^a^*P* values were calculated from unconditional logistic regression analysis.

^b^*P* values were calculated by unconditional logistic regression analysis with adjustments for age and gender.

*P ≤ 0.05 indicates statistical significance.

### Associations between haplotype analyses and IgAN risk

**Figure 1:**
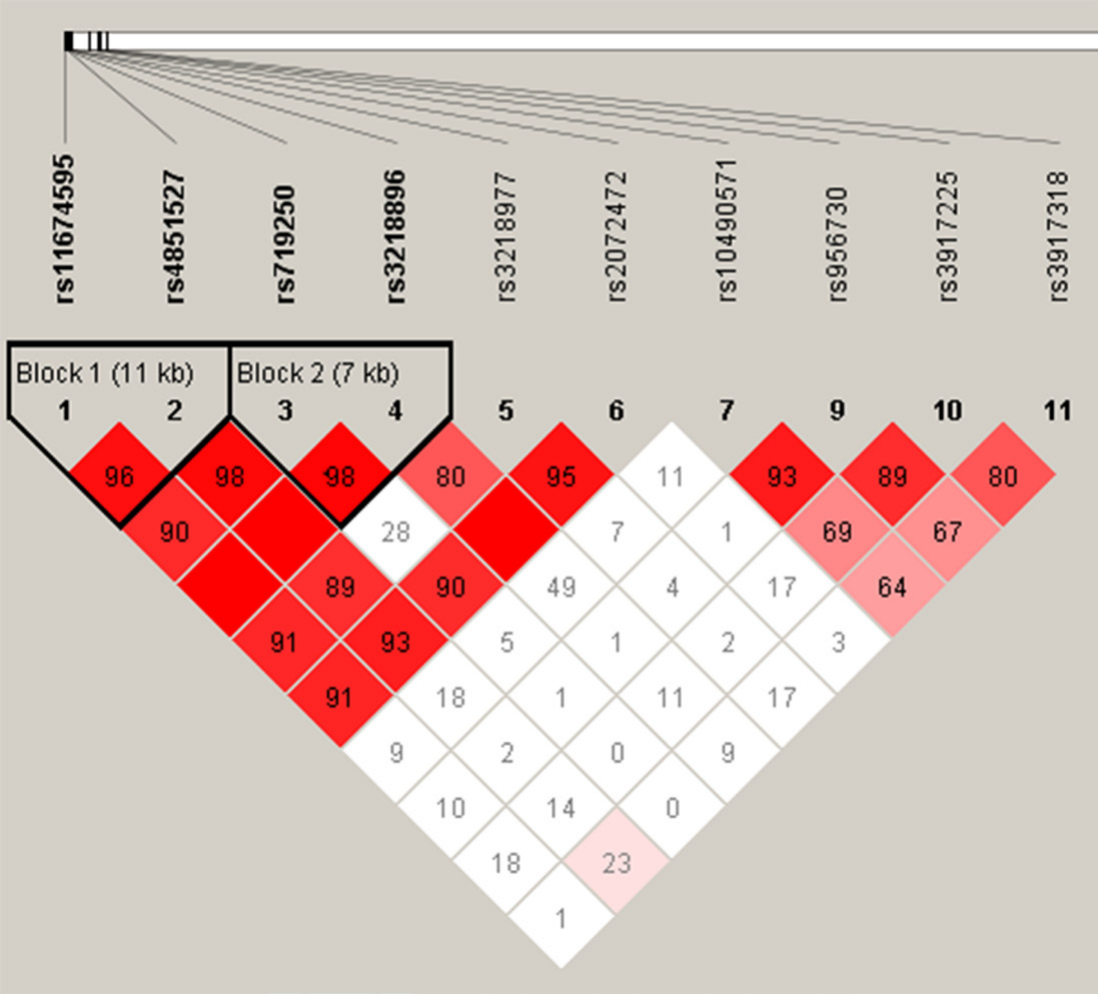
Linkage disequilibrium (LD) plots containing four SNPs from *IL1R2*

LD and haplotype analyses of the SNPs in the case and control samples were further studied. However, the four candidate SNPs in the *IL1R2* gene have showed strong linkage (Figure 1). We found that none of these SNPs showed evidence of interaction with age or gender of IgAN patients. As is shown in Table 4, the result for the IL1R2 haplotype was not found to be associated with a risk of IgAN, because the *p* value have no statistical difference. In addition, we have not found any association between *IL1R1* haplotype and the risk of IgAN.

**Table 4:** Haplotype analysis results in this study

SNPs	haplotype	Freq	without adjusted		with adjusted	
			OR (95% CI)	*P*	OR (95% CI)	*P*^a^
rs11674595/rs4851521	TG	0.493	1	---	1	---
	TA	0.298	1.13 (0.91–1.40)	0.27	1.18 (0.90–1.54)	0.23
	CG	0.207	1.00 (0.78–1.28)	0.98	1.04 (0.76–1.43)	0.8
rs719250/rs3218896	CT	0.686	1	---	1	---
	TT	0.157	1.00 (0.76–1.30)	0.97	1.05 (0.75–1.46)	0.78
	TC	0.156	1.01 (0.78–1.31)	0.94	0.95 (0.68–1.32)	0.76

Abbreviations: CI = confidence interval; OR = odds ratio; SNP = single nucleotide polymorphism.

P^a^: Adjusted by gender and age.

## DISCUSSION

Based on results from recent GWAS and previous studies, we chose to analyze 11 tSNPs in chromosomes 2 in our case control study. Using genetic model analysis, we found that two tSNPs (rs4851527, rs10490571) were associated with an increasing risk of IgAN. These results suggest that polymorphisms in these cytokine genes may play an important role in the risk of IgAN in the Han Chinese population.

Interleukin-1 (IL-1), as a family of multifunctional proinflammatory cytokine, plays a key role in autoimmune and inflammatory diseases by activating the expression of genes associated with the innate and adaptive immune response [10]. The IL-1 superfamily comprises the agonists *IL-1α* and *IL-1β* (predominant form in humans), and their antagonist *IL-1Ra* and both *IL-1* agonists can bind to *IL-1* receptor type 1(*IL-1R1*) and the “decoy” receptor IL-1 type 2 (*IL-1R2*) [18, 19]. *IL1R1*,as a Protein Coding gene, is located in a cluster of related cytokine receptor genes on chromosome 2q12,which belongs to the interleukin-1 receptor family and encodes a cytokine receptor [20]. It is an important mediator involved in many cytokine-induced immune and inflammatory responses [21]. *IL1R1* resulting from the SNPs (rs10490571 and rs3917225) may alter the combination of *IL1R1* and *IL-1* on the cell surface significantly and hence modulate the inflammatory processes associated with glomerular mesangial area destruction accordingly. This assumption was in accordance with previous findings that the degree of *IL1R1* expression on the cell surface affected the response of cells to *IL-1* [22]. Recent studies revealed that *IL1R1* expression was observably increased in several types of disease, including knee osteoarthritis [11], Prostate Carcinoma [23], hand osteoarthritis [24] and Inflammatory Bowel Disease [25]. However, we have not found any evidence for the role of heredity between *IL1R1* and IgAN susceptibility in previous studies. In our case-control study, we found that rs10490571, as the intronic SNP within the *IL1R1* gene, were markedly associated with IgAN risk according to both genotype and allele association analysis in a Chinese population. Based on the result of the study, the genotype of rs10490571 predicted an increased 1.46-fold and 1.36-fold IgAN risk, Therefore, this result indicate that *IL1R1* may play a pivotal role in IgAN and more samples and functional test are require to confirm our result.

*IL-1R2*, as a decoy receptor, is located on 2q11.2 in the human genome, which is principally expressed by neutrophils, B-cells, monocytes, and macrophages [26]. *IL1R2* is known to be a molecular decoy that sequesters *IL-1b*, and blocks the initiation of downstream signaling, thereby preventing inflammation. The *IL-1R2* receptor as a decrease of inflammation in several complex diseases, such as Ankylosing spondylitis [27], Arthritis [28],atherosclerosis [29] and endometriosis [30]. However, study based on this *IL1R2* gene polymorphism is rare and have no report association between the SNP *IL1R2* and IgAN risk. As part of this study, the GA genotype of rs4851527 exhibited increase IgAN risk. But the GA genotype of rs3218977 showed decrease IgAN risk. Hence, *IL1R2* gene may play an essential function in affecting IgAN. But the distinct role of *IL-1R2*, especially in IgAN, remains unknown and is worth our further research.

Some potential limitations of our current study should be considered when decipher the results. Firstly, we only included 426 IgAN patients and 463 health controls, so the sample size of our study may be small. Secondly, selection bias was inevitable. Thirdly, both IgAN patients and controls were gathered from a single hospital and therefore it may not be representative of the common population. Finally, associations between *IL1R1* and *IL1R2* polymorphisms and clinicopathological disease type were not evaluated in this study. Additional studies are needed to illuminate the genetic mechanisms underlying IgAN by fine-mapping the susceptibility regions of the variants.

To sum up, In our study, we confirmed two genes (*IL1R1* and *IL1R2*) are associated with risk of IgAN in Han Chinese population for the first time, which may provide new data to facilitate earlier diagnosis and promote early prevention, and shed light on the new candidate genes and new ideas for the study of subsequent occurrence mechanism of IgAN. Therefore, more studies should investigate these SNPs using more clinical data with bigger samples.

## MATERIALS AND METHODS

### Ethics statement

The study protocol was approved by the ethics committee of the First Affiliated Hospital of Xi'an Jiaotong University. Written informed consent was obtained from all participants after a full explanation of the study. The experimental protocol was implemented in accordance with the approved guidelines.

### Subjects

We recruited a total of 426 patients, which were diagnosed with IgAN (148 female and 278 male, mean age of 33+12.1 years)by renal biopsy were enrolled from the First Affiliated Hospital of Xi'an Jiaotong University from March 2011 to April 2016. The controls were 463 healthy subjects (265 males and 198 females, mean age of 50 ± 11.8 years) recruited from routine healthy examinations in the same hospitals. All subjects were from the Chinese Han population living in Xi'an. All the patients were recently diagnosed and histologically confirmed to suffer from IgAN according to the renal biopsy, and they had not received any systemic treatment before the time of examination. Besides, patients with cancer, infection, secondary IgAN (Secondary IgAN is seen most commonly in patients with liver disease or mucosal inflammation, in particular affecting the gastrointestinal tract), other renal diseases and autoimmune diseases were excluded. The exclusion criteria for healthy subjects included the chronic disease, central nervous system-related disease, and conditions involving vital organs (liver, heart, lung, brain) and more aggressive metabolic and endocrinological disease. SNP selection and genotyping

All 11 SNPs in the *IL1R1* and *IL1R2* gene with minor allele frequencies > 5% in the HapMap (http://www.hapmap.org) Han Chinese population. Blood samples were collected in tubes containing ethylene diaminetetraacetic acid (EDTA) and stored at −80C after centrifugating at 1,500 rpm for 10 min. Genomic DNA from whole blood was extracted using the GoldMag DNA Purification Kit (GoldMag Co. Ltd, Xi’an City, China), and the purity and concentration was measured utilizing an ultraviolet spectrophotometer (Nanodrop, Thermo Scientific, Waltham, MA). The Sequenom MassARRAY Assay Design 3.0 software was used to design Multiplexed SNP MassEXTEND assay. SNP genotyping was performed by using Sequenom MassARRAY RS1000 according to the standard protocol. Sequenom Typer 4.0 software was used for data management and analysis.

### Statistical analysis

We performed Statistical analyses was using Microsoft Excel and SPSS 16.0 (SPSS, Chicago,IL, USA) to In this study, all *p* values were two-sided (*p* ≤ 0.05 was considered as achieving the threshold of statistical significance). Each SNP frequency in the control subjects was tested for deviation from Hardy–Weinberg equilibrium (HWE) by the Fisher's test. Calculate the genotype and allele frequencies in patients and controls were also compared using the χ^2^ test. Odds ratio (OR) values and 95% confidence intervals (CIs) measured risk allele effect size using unconditional logistic regression analysis [31]. Five genetic models (codominant, dominant, recessive, overdominant, and log-additive) were used to evaluate potential association of *IL1R1* and *IL1R2* polymorphisms with risk and clinical parameters of IgAN. Statistical analyses were performed using Microsoft Excel and the SPSS 17.0 statistical package (SPSS Inc, Chicago, Illinois, USA). Finally, the Haploview were used to construct haplotype and genetic association at significant polymorphism loci and to estimate the pairwise linkage disequilibrium (LD), haplotype constru software (version4.2) and SHEsis software platform (http://www.nhgg.org/analysis/) ction, and genetic association at polymorphism loci [32, 33].
